# Student Preferences for Feedback Delivery in Healthcare Education: A Cross-Sectional Survey

**DOI:** 10.7759/cureus.91442

**Published:** 2025-09-01

**Authors:** Pei Nee Wong, Pei Se Wong

**Affiliations:** 1 Pharmacy Department, Fatima College of Health Sciences, Abu Dhabi, ARE; 2 Pharmacy Department, IMU University, Kuala Lumpur, MYS

**Keywords:** clinical education, educational assessment, feedback preferences, health professions education, student learning

## Abstract

Introduction: Feedback plays a vital role in student learning by identifying areas for improvement and promoting academic growth. The effectiveness of feedback depends on its form and when it is delivered. The literature has shown that students express a desire for more systematic and timely feedback and diverse strategies for feedback delivery. However, little is known about healthcare profession students' preferred feedback styles in different learning activities.

Purpose: The study aims to investigate the preferred feedback styles of pharmacy and nursing students in Fatima College of Health Sciences (FCHS), a healthcare education institution in the United Arab Emirates.

Methods: A web-based survey was sent to pharmacy and nursing students on two campuses. The survey assessed preferred feedback methods in six areas: preparedness, performance, and attitude during a clinical activity, technical procedures, and inpatient and outpatient care. Respondents chose their preferred feedback method for each area.

Results: A total of 196 students completed the survey. Uniquely, the findings showed that the majority of students valued feedback on preparation, performance, attitude, and patient care most when it was given privately after the activity, whereas feedback on technical procedures was better received when delivered immediately and openly during the activity.

Conclusion: The findings revealed the need to tailor feedback approaches to both task type and learner expectations. These insights can be leveraged to enhance teaching practice and guide faculty training, thereby strengthening feedback practices in healthcare professions education.

## Introduction

Feedback is essential for learning, particularly in healthcare education, where students must develop both theoretical knowledge and practical skills [1]. Appropriate feedback can significantly contribute to developing learners’ competence and confidence, helping them to identify gaps between their actual and desired performance and find methods for improvement [2]. With educational focus in the healthcare field shifting toward competency-based approaches, feedback has become more crucial in ensuring students reach their specific learning milestones and develop the necessary skills to meet healthcare needs [3].

Several studies have explored the impact of feedback on student learning in health professions programmes. Ahmady et al. [4] and Alfehaid et al. [5] both emphasised the importance of continuous, constructive feedback with balanced positive comments and points for improvement. Al-Mously and colleagues [6] identified significant challenges in providing and utilising feedback to medical students, including a lack of effective feedback and the need for improved feedback training. Educators frequently offered performance evaluations and task descriptions but were less likely to clarify session purpose or promote learner involvement [7].

Various factors have been identified to enhance feedback effectiveness, including establishing an appropriate interpersonal climate, offering specific feedback, and using non-judgmental feedback [8,9]. Research supports the notion of differentiating between various forms of feedback: direct feedback (i.e., explicit and straightforward feedback), indirect feedback (i.e., implicit and subtle), specific feedback (i.e., detailed on performance), and group-based and individualised feedback [10,11]. Feedback timing, such as providing feedback immediately after an event or later, can significantly affect the effectiveness of feedback in promoting learning and improvement. Several studies advocated for the benefits of immediate feedback [12,13]. Research has found evidence for the “delay-retention effect,” indicating that delayed feedback can lead to improved learning outcomes [14,15], particularly for complex or difficult questions. However, students desired more systematic and timely feedback, with a preference for written comments and item difficulty feedback [9]. Research underscores the necessity of acknowledging the learning style preferences of students and allied health practitioners, respectively, when deciding on feedback methods [16-18].

In the clinical workplace, feedback is widely recommended to be given in private [19]. Sociocultural factors such as feedback culture, trust, and student agency influenced how feedback is perceived and used. This suggests that exposure to a real-world clinical environment could influence students’ preferences and engagement with feedback depending on the context and culture of learning environments.

There is limited knowledge regarding health professions students’ preferences for different feedback methods and whether the learning environment, such as prior exposure to clinical placements, influences these preferences. To address this gap, the present study investigated pharmacy and nursing students’ preferences for feedback styles during clinical activities, focusing on whether they favoured private or group feedback across different aspects of learning, including preparation, performance, attitude, and patient care. We hypothesised that pharmacy and nursing students would demonstrate a stronger preference for receiving feedback in private rather than in group settings. The findings can inform instructional design and feedback practices as educators seek to optimise learning outcomes for students with diverse learning preferences and needs.

## Materials and methods

Study design and setting

This cross-sectional study was conducted between April and May 2024 at Fatima College of Health Sciences (FCHS) across two campuses (Abu Dhabi and Al Ain). The Fatima College of Health Sciences Institutional Ethics Review Board (ref. number: FECE-2-23-24-Wong2) granted ethical approval for the study.

Study participants and setting

The target participants comprised 735 undergraduate pharmacy and nursing students enrolled in the academic year of 2024-2025 at Fatima College of Health Sciences, located in the UAE. Student contact information was retrieved from institutional records, and potential participants were invited to the study via an email containing a link to the online questionnaire. A convenience sampling approach was adopted, targeting the students enrolled in the pharmacy and nursing programs during the study period.

The setting institution offers a five-year undergraduate pharmacy program and a four-year undergraduate nursing program. Both programmes integrate academic learning with progressive clinical placements, which begin in the second year and increase in frequency and complexity in the final years. These placements provide students with hands-on experience under supervision by clinical instructors. The curriculum for the pharmacy programme focuses on pharmacology, medication management, and patient counselling, while the nursing programme curriculum emphasises direct patient care, clinical procedures, and health education.

Study instrument and distribution

The data were collected using a questionnaire administered to the students through SurveyMonkey®. Student participants were asked to fill out two sections: section A, which included demographical data, consisting of information on age, area of study, level of study, campus registered to, and whether they attended clinical placement; and section B, which comprised a modified 6-item questionnaire utilised by a previous study [11]. Prior permission to use and adapt the tool was obtained from the original author. To ensure relevance and clarity of the questionnaire, face and content validity were established by five pharmacy faculty members and the instrument was subsequently piloted with five students. Following validation, minor modifications were made to the questionnaire to enhance clarity and contextual relevance. The original question was changed to improve clarity and make it more relatable for students. The term "clinical activity" replaced "high-stakes team activity" because it is a more straightforward and understandable term for students. In addition, "intern" and "instructor" were used instead of "a member" and "your superior” to better reflect the context of students’ clinical placements. These modifications did not alter the underlying constructions or measurement properties of the instrument.

The final version of the questionnaire (see Table 1) evaluates the feedback methods throughout clinical activities across six domains: preparedness, performance, attitude, technical procedures, and inpatient and outpatient care. For each domain, participants indicated their preferred type and timing of feedback by selecting one of the following responses: 1) during the activity, direct specific feedback, in front of the team; 2) during the activity, indirect specific feedback, in front of the team; 3) after the activity, direct specific feedback, away from the team; 4) after the activity, indirect specific feedback, away from the team; 5) during a scheduled review, aggregated feedback, in front of the team; or 6) during a scheduled review, aggregated feedback, away from the team.

**Table 1:** Survey Items

Question: You are an intern in a healthcare team. While performing a clinical activity, your instructor is disappointed with you in the following domain. Which feedback method would you prefer? Note: Aggregated feedback refers to a collection of feedback from various sources.						
Domain	(1) during the activity, direct specific feedback, in front of the team	(2) during the activity, indirect specific feedback, in front of the team	(3) after the activity, direct specific feedback, away from the team	(4) after the activity, indirect specific feedback, away from the team	(5) during a scheduled review, aggregated feedback, in front of the team; or	(6) during a scheduled review, aggregated feedback, away from the team.
Preparedness						
Performance						
Attitude						
Technical procedures						
Inpatient care						
Outpatient care						

Statistical analyses

Participants’ demographics were described using frequency statistics. Feedback preferences in each of the six domains were compared based on subgroups (pharmacy and nursing). Content validity was established through feedback from faculty and students. Content Validity Index (CVI) was not calculated, as the questionnaire was adapted from previously validated instruments. Reliability in this study was assessed using Cronbach’s alpha. Chi-square analysis was employed to determine whether feedback preference distributions differed between subgroups.

## Results

Participants' demographic details

One hundred ninety-six participants completed the survey, with an overall response rate of 26.7%. There was a 52.6% response rate for pharmacy students, compared to 47.4% for nursing students. Table 2 presents the participant characteristics. The majority of the respondents were female (98.5%), and most of them registered in Year 3 (28.1%). The reliability of the six-item questionnaire was assessed using Cronbach's alpha. The scale demonstrated excellent internal consistency, with a Cronbach's alpha coefficient of 0.887, indicating a high degree of reliability for the instrument in measuring feedback methods across the six domains.

**Table 2:** Sociodemographic characteristics of the participants

Characteristics	Pharmacy (n = 103)	Nursing (n = 93)	Overall (n = 196)
Gender			
Female	103 (52.6)	90 (45.9)	193 (98.5)
Male	0	3 (1.5)	3 (1.5)
Campus			
Abu Dhabi	68 (43.7)	49 (25.0)	117 (59.7)
Al Ain	35 (17.9)	44 (22.4)	79 (40.3)
Level of study			
Year 1	11 (5.6)	20 (10.2)	31 (15.8)
Year 2	8 (4.1)	35 (17.9)	43 (21.9)
Year 3	35 (17.9)	20 (10.2)	55 (28.1)
Year 4	31 (5.8)	18 (9.2)	49 (25.0)
Year 5	18 (9.2)	0	18 (9.2)
Have you attended clinical placement?			
Yes	84 (42.9)	53 (27.0)	137 (69.9)
No	19 (9.7)	40 (20.4)	59 (30.1)

Preferred Feedback Among Pharmacy and Nursing Students

**Figure 1:**
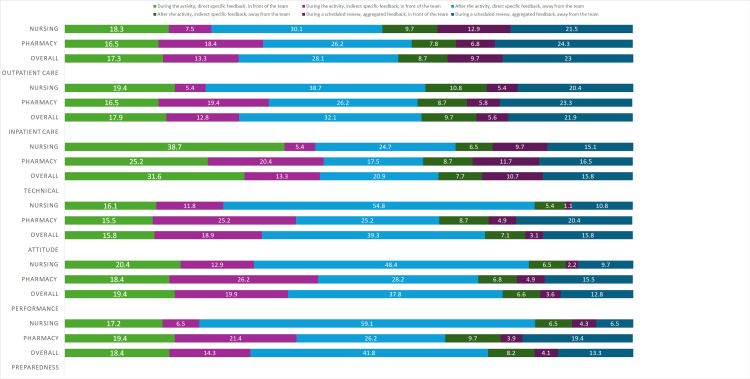
Preferred feedback among pharmacy and nursing students.

Figure 1 presents data on the preferred feedback styles among nursing and pharmacy students across various domains. Feedback “after the activity, direct specific feedback, away from the team” across all domains is the most preferred by the majority of students, particularly among nursing students in the preparedness domain (59.1%), except for technical procedures. For technical procedures, most students preferred feedback “during the activity, direct specific feedback, in front of the team.” Nursing students exhibited a higher preference for this style (38.7%) compared to pharmacy students (25.2%). This suggests that while students generally prefer private feedback, they may be more open to public feedback in contexts requiring immediate evaluation of technical skills.

Feedback “after the activity, indirect specific feedback, away from the team” or “during a scheduled review, aggregated feedback, in front of the team” are least preferred across all domains. Aggregated feedback in front of the team, particularly during a scheduled review session, ranks as the least preferred, with only 1.1% of nursing students and 4.9% of pharmacy students selecting it in the attitude domain, and an overall 3.6% of students in the performance domain.

Preferred Feedback Between Students Who Have Attended and Never Attended Placement

Table 3 presents data on the preferred feedback methods among students who attended a placement and those who did not. A chi-square test of independence was carried out to examine the relationship between clinical placement attendance and their choice of feedback styles in each domain. The relation between the two variables was insignificant in all domains (p > 0.05).

**Table 3:** Preferred feedback between students who have attended vs. those who never attended placement.

Domain	(1) during the activity, direct specific feedback, in front of the team	(2) during the activity, indirect specific feedback, in front of the team	(3) after the activity, direct specific feedback, away from the team	(4) after the activity, indirect specific feedback, away from the team	(5) during a scheduled review, aggregated feedback, in front of the team; or	(6) during a scheduled review, aggregated feedback, away from the team.		
Preparedness								
Attended	22 (16.1%)	19 (13.9%)	56 (40.9%)	13 (9.5%)	7 (5.1%)	20 (14.6%)	p = 0.515	
Never	14 (23.7%)	9 (15.3%)	26 (44.1%)	3 (5.1%)	1 (1.7%)	6 (10.2%)		
Performance								
Attended	22 (16.1%)	28 (20.4%)	53 (38.7%)	10 (7.3%)	5 (3.6%)	19 (13.9%)	p = 0.619	
Never	16 (27.1%)	11 (18.6%)	21 (35.6%)	3 (5.1%)	2 (3.4%)	6 (10.2%)		
Attitude								
Attended	18 (13.1%)	30 (21.9%)	52 (38.0%)	9 (6.6%)	6 (4.4%)	22 (16.1%)	p = 0.201	
Never	13 (22.0%)	7 (11.9%)	25 (42.4%)	5 (8.5%)	0 (0.0%)	9 (15.3%)		
Technical procedures								
Attended	45 (32.8%)	18 (13.1%)	29 (21.2%)	13 (9.5%)	12 (8.8%)	20 (14.6%)	p = 0.509	
Never	17 (28.8%)	8 (13.6%)	12 (20.3%)	2 (3.4%)	9 (15.3%)	11 (18.6%)		
Inpatient care								
Attended	23 (16.8%)	18 (13.1%)	46 (33.6%)	15 (10.9%)	6 (4.4%)	29 (21.2%)	p = 0.742	
Never	12 (20.3%)	7 (11.9%)	17 (28.8%)	4 (6.8%)	5 (8.5%)	14 (23.7%)		
Outpatient care								
Attended	23 (16.8%)	17 (12.4%)	41 (29.9%)	15 (10.9%)	10 (7.3%)	31 (22.6%)	p = 0.276	
Never	11 (18.6%)	9 (15.3%)	14 (23.7%)	2 (3.4%)	9 (15.3%)	14 (23.7%)		

## Discussion

Building upon the established importance of feedback in the learning process [1], this study examined the feedback preferences of healthcare profession students in a UAE institution. The findings revealed a clear preference for specific, private feedback provided post-activity completion. Public or aggregated feedback, particularly when indirect, was less favoured by the majority of students.

This is in line with findings of Pascarella et al. [11], where the researched students preferred receiving public feedback on their technical performance in front of team members during active engagement with the activity. This preference might be attributed to the objective nature of technical skills, making them more easily assessed and measured [20]. Moreover, students typically find it easier to understand and accept feedback delivered publicly when it is directly linked to observable performance in technical tasks.

Feedback on preparedness, performance, attitude, and patient care encompasses a range of professional attributes and clinical skills, including communication, empathy, and patient handling, which are often considered more subjective and challenging to assess [21,22]. The preference for private feedback post-activity may be attributed to the desire to process potential criticism without the anxiety of public exposure, which can potentially harm self-esteem [23]. Moreover, personalised feedback, which is often more familiar to students, is recognised for its effectiveness in addressing individual student needs and learning gaps [10], providing tailored advice for improvement. While students who attended placement showed a stronger inclination for direct, private feedback after the activity, this difference was not statistically significant between students who attended placement and those who did not.

When given a choice between specific and aggregated feedback (i.e., feedback summarised across a group), students demonstrate a preference for specific feedback. This preference also aligns with the findings of Perera et. al [24], which highlight the significance of specific feedback in facilitating effective learning. Although aggregated feedback can identify general trends, its lack of specificity, actionability, and personal connection may limit its perceived value for individual student growth. Combining “specific” and “aggregated” feedback would potentially help suggest specific resources or activities that can help students master a specific competence. 

Evidence suggests that the optimal timing of feedback varies based on the task’s nature and complexity. While immediate feedback may be beneficial for short-term learning and procedural skills development [25], more complex tasks may require greater processing time. Delayed feedback allows for this reflection. For tasks requiring in-depth processing, delayed feedback can enhance learning and performance outcomes [1]. This finding aligns with our observation that most students preferred feedback during clinical activities involving diverse skills and attributes, likely reflecting the need for deeper reflection and integration.

Students undoubtedly have diverse preferences for feedback, which are influenced by various factors. These factors include their perception of the task itself (e.g., novelty, difficulty, and stakes), the nature of the feedback (e.g., positive/negative, task- vs. person-focused, expected/unexpected), the feedback delivery method (e.g., private vs. public, scheduled vs. unscheduled), and individual student factors such as physiological, psychological, and affective states. For example, a student’s mood or level of motivation can significantly impact their receptiveness to feedback. Additionally, their perception of their own performance and the anticipated outcome of the assessment can influence their preferences. These findings underscore the importance of considering individual student needs and preferences when designing and delivering feedback.

Teachers and instructors must carefully consider the feedback method and timing to create an environment where students feel understood, respected, and actively engaged in the feedback process [26]. Beyond adapting feedback to task-specific student preferences into educators' teaching practices, the findings can be leveraged through faculty development to foster a stronger feedback culture. Faculty training can emphasise the strategies to balance the logistical constraints while honouring students’ needs for privacy. For instance, faculty training to focus on the brief feedback model, e.g. One-Minute Preceptor for the private individual feedback, and adopting group briefings using a method, e.g., SET-GO [27] to address broader learning points. Digital platforms (e.g., recorded audio feedback) can provide feedback asynchronously, reducing time pressures without compromising the sense of privacy. By aligning faculty development with learners’ preferences while recognising logistical realities, institutions can foster a feedback culture that is both responsive and sustainable.

Limitations

This study has various limitations. Since this study was conducted at a single institution, local practices and education culture may have influenced our participants’ perspectives and experiences [28]. Most participants were female, reflecting both the institution’s origins as a women’s college (admitting male students only since 2011) and the female predominance in nursing and pharmacy. The study achieved a relatively low response rate (26.7%), although the absolute number of participants of 196 was sufficient to provide meaningful insights into feedback preferences. Both factors limit the extent to which our findings can be generalised. Another limitation is the study’s reliance on self-reported data, which may introduce bias or inaccuracies due to students’ perceptions and recollections. The study’s cross-sectional design further hinders the ability to establish causality or monitor preference changes over time. Future research could address these limitations by employing longitudinal designs to track changes in students’ feedback preferences over time and by including multi-institutional or larger, more diverse samples to improve the generalizability of findings. This study was limited to exploring feedback preferences within a clinical setting, which allowed us to capture perspectives in a practice-oriented learning environment. Future research examining how different feedback approaches influence learning outcomes across varied educational contexts (e.g., classroom-based learning) would provide valuable insights.

## Conclusions

To our knowledge, this is the first study to explore healthcare students’ preferences for feedback styles within the UAE context. This study revealed distinct preferences for feedback styles among pharmacy and nursing students, with a general inclination towards private, direct feedback delivered after an activity, particularly for subjective performance areas like preparedness, attitude, and patient care. Conversely, students preferred direct, in-front-of-the-team feedback during technical procedures, suggesting that the objective nature of skills influences receptiveness to public feedback. The findings underscore the importance of tailoring feedback delivery to the specific activity and individual student needs, moving away from uniform approaches. Given that direct private feedback is often challenged by logistical constraints, faculty training should emphasise practical strategies and structured models that balance student needs with feasibility. This can enable institutions to foster a sustainable feedback culture that enhances learning and engagement in both clinical and educational settings.

## References

[1] Hattie J, Timperley H (2007). The power of feedback. Rev Educ Res.

[2] Hardavella G, Aamli-Gaagnat A, Saad N, Rousalova I, Sreter KB (2017). How to give and receive  feedback effectively. Breathe (Sheff).

[3] Ramani S, Krackov SK (2012). Twelve tips for giving feedback effectively in the clinical environment. Med Teach.

[4] Ahmady S, Zand S, Nikravan-Mofrad M (2015). Student satisfaction on getting feedback in clinical teaching. J Med Edu Dev.

[5] Alfehaid LS, Qotineh A, Alsuhebany N (2018). The perceptions and attitudes of undergraduate healthcare sciences students of feedback: a qualitative study. Health Prof Educ.

[6] Al-Mously N, Nabil NM, Al-Babtain SA, Fouad Abbas MA (2014). Undergraduate medical students' perceptions on the quality of feedback received during clinical rotations. Med Teach.

[7] Johnson CE, Keating JL, Farlie MK, Kent F, Leech M, Molloy EK (2019). Educators' behaviours during feedback in authentic clinical practice settings: an observational study and systematic analysis. BMC Med Educ.

[8] Hewson MG, Little ML (1998). Giving feedback in medical education: verification of recommended techniques. J Gen Intern Med.

[9] Bazrafkan L, Ghassemi Gh H, Nabeiei P (2013). Feedback is good or bad? Medical residents’ points of view on feedback in clinical education. J Adv Med Prof.

[10] Engerer C, Berberat PO, Dinkel A, Rudolph B, Sattel H, Wuensch A (2019). Specific feedback makes medical students better communicators. BMC Med Educ.

[11] Pascarella L, Marulanda K, Duchesneau ED, Sanchez-Casalongue M, Kapadia M, Farrell TM (2023). Preferred feedback styles among different groups in an academic medical center. J Surg Res.

[12] Clare L, Jones RS (2008). Errorless learning in the rehabilitation of memory impairment: a critical review. Neuropsychol Rev.

[13] Choi S, Oh S, Lee DH, Yoon HS (2020). Effects of reflection and immediate feedback to improve clinical reasoning of medical students in the assessment of dermatologic conditions: a randomised controlled trial. BMC Med Educ.

[14] Clariana RB, Wagner D, Roher Murphy LC (2000). Applying a connectionist description of feedback timing. Educ Technol Res Dev.

[15] Smith TA, Kimball DR (2010). Learning from feedback: spacing and the delay-retention effect. J Exp Psychol Learn Mem Cogn.

[16] Vittetoe MC, Hooker E (1983). Learning style preferences of allied health practitioners in a teacher education program. J Allied Health.

[17] Zoghi M, Brown T, Williams B (2010). Learning style preferences of Australian health science students. J Allied Health.

[18] Tasdemir MS, Yalcin Arslan F (2018). Feedback preferences of EFL learners with respect to their learning styles. Cogent Educ.

[19] Fuentes-Cimma J, Sluijsmans D, Riquelme A, Villagran I, Isbej L, Olivares-Labbe MT, Heeneman S (2024). Designing feedback processes in the workplace-based learning of undergraduate health professions education: a scoping review. BMC Med Educ.

[20] Castillo-Segura P, Fernández-Panadero C, Alario-Hoyos C, Muñoz-Merino PJ, Delgado Kloos C (2021). Objective and automated assessment of surgical technical skills with IoT systems: a systematic literature review. Artif Intell Med.

[21] Ten Cate O, Regehr G (2019). The power of subjectivity in the assessment of medical trainees. Acad Med.

[22] Virk A, Joshi A, Mahajan R, Singh T (2020). The power of subjectivity in competency-based assessment. J Postgrad Med.

[23] Mercer M, Gulseren DB (2023). When negative feedback harms: a systematic review of the unintended consequences of negative feedback on psychological, attitudinal, and behavioral responses. Stud High Educ.

[24] Perera J, Lee N, Win K, Perera J, Wijesuriya L (2008). Formative feedback to students: the mismatch between faculty perceptions and student expectations. Med Teach.

[25] Schroth M (1992). The effects of delay of feedback on a delayed concept formation transfer task. Contemp Educ Psychol.

[26] Hausman M, Detroz P, Pétré B (2023). Feedback processing and emotion regulation in nursing students during internship. Learn Instr.

[27] Orsini C, Rodrigues V, Tricio J, Rosel M (2022). Common models and approaches for the clinical educator to plan effective feedback encounters. J Educ Eval Health Prof.

[28] Ramani S, Post SE, Könings K, Mann K, Katz JT, van der Vleuten C (2017). "It's just not the culture": a qualitative study exploring residents' perceptions of the impact of institutional culture on feedback. Teach Learn Med.

